# Case of Compound Dislocation of the Knee Joint

**Published:** 1848-05

**Authors:** O. H. Costill

**Affiliations:** Frankford, Pennsylvania


### *Case of Compound Dislocation of the Knee Joint.* By O. H. Costill, M. D., of Frankford, Pennsylvania.

On the 5th of November last, I was called to see E. B., a boy aged eleven years, said to have his leg broken; the accident had occurred rather more than an hour previous to my seeing him. I found him lying on a settee, in great suffering. Upon ripping up the left leg of his pantaloons, I found the head of the tibia completely exposed, having been thrust entirely through the soft parts covering the joint, posteriorly and a little outwards. As nearly as could be ascertained, the accident occurred in the following manner. The boy suspended himself from the back part of a wagon which was passing through the street; in the act of extending his legs beneath the wagon, the left one was caught between the spokes of the wheel, which, by its rotation, brought the anterior part of the limb in contact with the wagon; the boy still clinging (through fright) to his first position, the force thus applied, as the bone did not break, drove the head of it backwards and produced the displacement stated above.

By making extension and at the same time flexing the leg, while the body was firmly held, I reduced the luxation without difficulty, though some hemorrhage attended. The laceration was severe; the whole of the posterior part of the joint was exposed, and the popliteal artery distinctly visible. I brought the integuments together by adhesive strips and stitches, and removed with the scissors some shreds of tendon which hung from the wound.

A compress and roller were then lightly applied, and the limb was supported in a slightly flexed position by a splint on the inside, extending from the foot nearly to the groin; a pillow was placed under the knee. The patient was directed to take a teaspoonful of paregoric, to be repeated during the night if necessary to procure rest. It was 7 o'clock, P. M., when the dressing was finished. The pulse and skin were natural, and the boy expressed himself as feeling much more comfortable than before the limb was dressed; nevertheless I thought it right to apprise his parents of the dangerous nature of the accident. The next morning at six o'clock I saw him again, he had complained much during the night, but had some sleep, and was as well as if the case had been one of ordinary fracture. The dressings were saturated by discharge from the wound. In the evening Dr. Taylor saw the patient with me; at this time there was some fever, the pulse however, did not exceed 100; we directed an infusion of senna, and the anodyne to be repeated. Nov. 7th. He is much more comfortable this morning; no unfavourable symptom has occurred. On the fifth day, for the first time, the dressings were removed. Suppuration had partially commenced. A poultice of bread and milk was applied, secured by a many-tailed bandage. The splint was continued, and the limb rested on a pillow. In this way the dressings were renewed daily, with but little variation; free suppuration and some sloughing occurred, but the wound healed in six weeks, and in two weeks more the boy could walk with slight assistance. He is now perfectly well, except that the joint admits of but little motion, although not entirely anchylosed.

*March 1st*, 1848.

